# Palliative and Supportive Care Needs in Advanced Hidradenitis Suppurativa: A Systematic Review

**DOI:** 10.7759/cureus.108573

**Published:** 2026-05-09

**Authors:** Muhammad A Almahdi, Amany Mashi, Ali Y Shaikh, Mayar M Alahmadi, Meshari A Alalyani, Manar S Alghamdi, Shorooq A Hamzi

**Affiliations:** 1 Faculty of Medicine, Jazan University, Jazan, SAU; 2 Department of Dermatology, Armed Forces Hospital, Jazan, SAU; 3 College of Medicine and Surgery, Umm Al-Qura University, Makkah, SAU; 4 College of Medicine, King Saud Bin Abdulaziz University for Health Sciences, Jeddah, SAU; 5 Faculty of Medicine, Umm Al-Qura University, Makkah, SAU

**Keywords:** hidradenitis suppurativa, palliative care, quality of life, supportive care, unmet needs

## Abstract

Hidradenitis suppurativa (HS) is a chronic inflammatory skin condition associated with severe symptom burden, high psychiatric comorbidity, and profound impairment in health-related quality of life (HRQoL). Despite the disease burden, no systematic synthesis has characterized the palliative and supportive care needs of this population. This review aimed to synthesize evidence on the palliative and supportive care needs of adults with advanced HS, to evaluate validated patient-reported outcome measures applicable to this domain, and to characterize unmet needs and barriers to care. This systematic review was conducted in accordance with Preferred Reporting Items for Systematic Reviews and Meta-Analyses (PRISMA) 2020 guidelines and registered on the International Prospective Register of Systematic Reviews (PROSPERO). Studies reporting palliative care-relevant outcomes in adults with HS were included. Quality was appraised using Joanna Briggs Institute (JBI) Prevalence Checklists, Newcastle-Ottawa Scale, and Critical Appraisal Skills Programme (CASP) tools. Seven studies (sample sizes ranging from 77 to 1,787; conducted across 27 countries; published between 2020 and 2025) were included. Across included studies, 61.4% (n = 798/1,299) of the patients experienced moderate-to-severe pain, while drainage and fatigue were reported by 71.8% (n = 933/1,299) and 61.0% (n = 793/1,299), respectively. Depression was present in 30.3-35.8% of participants (from 154/508 to 465/1,299) and anxiety in 36.2-57.5% (from 470/1,299 to 292/508); suicidal ideation was reported by 7.9% (n = 103/1,299) and suicidal attempt by 4.2% (n = 55/1,299). Sexual dysfunction affected 60.8% (n = 45/74), and hindered sexuality affected 61.8% (n = 47/76). Only 4.5% (n = 24/529) of adults achieved complete disease control despite active dermatologist treatment. Emotional burden consistently exceeded physical symptom burden. Caregiver needs, spiritual concerns, and advance care planning remain unstudied. Adults with advanced HS carry a multidimensional, unmet palliative and supportive care burden, underscoring the importance of integration of psychological screening, sexual health assessment, and pain management protocols.

## Introduction and background

Hidradenitis suppurativa (HS) is a chronic, relapsing inflammatory skin condition. It is characterized by recurrent painful nodules, abscesses, sinus tracts, and scarring in intertriginous skin regions. Recent meta-analyses estimate HS prevalence at 0.3%-0.40% [1,2]. HS is linked to a broad range of comorbidities. These include metabolic syndrome, inflammatory bowel disease, and elevated cardiovascular risk, and a systemic inflammatory phenotype, causing substantial morbidity [3,4]. Psychiatric sequelae are severe. Depression and anxiety rates are higher than in the general population [5]. Completed suicide rates are significantly increased [6]. The impact on health-related quality of life (HRQoL) exceeds that of moderate-to-severe psoriasis [7].

Palliative care means improving quality of life (QoL) by preventing and easing physical, psychosocial, and spiritual suffering [8]. This definition now includes serious chronic conditions recognized by persistent symptoms, progressive functional decline, and high psychiatric comorbidity [9]. Expert consensus defines palliative care eligibility by three criteria: persistent symptoms not fully relieved by disease-modifying therapy, significant psychological morbidity, and progressive disability. Advanced HS, defined as Hurley Stage II or III disease (or equivalent to International Hidradenitis Suppurativa Severity Score System (IHS4) ≥11), is characterized by interconnecting sinus tracts, scarring, and recurrent abscesses that are refractory to standard pharmacological therapy. Advanced HS fulfils each criterion for palliative care eligibility: (1) persistent physical symptoms, most notably pain, drainage, and fatigue, that are not fully relieved by available disease-modifying therapies; (2) substantial psychological morbidity, including depression, anxiety, and suicidal ideation; and (3) progressive functional and occupational disability that accumulates with disease duration irrespective of treatment. These criteria collectively constitute a palliative care imperative that current HS clinical frameworks do not formally address.

Several dimensions of care needs are particularly poorly addressed: pain management lacks validated protocols; psychological and psychiatric burden is rarely formally screened; sexual health is rarely assessed in dermatological practice; and the diagnostic delay represents a period of unrecognized and untreated suffering. To date, no systematic synthesis has examined palliative and supportive care needs in HS. This review addresses that gap by (1) synthesizing evidence on physical, psychological, social, sexual, and informational care needs in adults with advanced HS; (2) evaluating validated patient-reported outcome measures applicable to this domain; and (3) characterizing unmet needs, barriers, and research priorities.

## Review

Methods

Protocol and Registration

This review was conducted and reported in accordance with the Preferred Reporting Items for Systematic Reviews and Meta-Analyses (PRISMA) 2020 guidelines [10] and prospectively registered on the International Prospective Register of Systematic Reviews (PROSPERO) (registration number: CRD420261360950).

Eligibility Criteria

Adults (≥18 years) with physician-confirmed HS and reporting outcomes relevant to palliative or supportive care, including physical symptom burden, psychiatric morbidity, social and occupational functioning, sexual health, HRQoL, medical care access, or treatment satisfaction, were eligible. Studies were required to include a subgroup or majority with Hurley Stage II or III, or equivalent markers of advanced disease (e.g., IHS4 ≥11). Studies exclusively enrolling children, focused solely on biologic efficacy without palliative care outcomes, or comprising case reports were excluded. The complete inclusion and exclusion criteria are presented in Table 1.

**Table 1:** Inclusion and exclusion criteria HS, hidradenitis suppurativa; HRQoL, health-related quality of life; IHS4, International Hidradenitis Suppurativa Severity Score System

Criterion Type	Criterion
Inclusion	Adults aged ≥18 years
	Physician-confirmed diagnosis of hidradenitis suppurativa (HS)
	Reporting outcomes relevant to palliative or supportive care (physical symptom burden, psychiatric morbidity, social and occupational functioning, sexual health, HRQoL, medical care access, or treatment satisfaction)
	Studies including a subgroup or majority with Hurley Stage II or III, or equivalent markers of advanced disease (e.g., IHS4 ≥11)
	Published in English in a peer-reviewed journal
Exclusion	Studies exclusively enrolling children or adolescents (<18 years)
	Studies focused solely on biologic efficacy without palliative or supportive care outcomes
	Case reports
	Conference abstracts without full-text availability
	Non-English language publications

Search Strategy

Searches were performed across MEDLINE, Web of Science, and Cochrane CENTRAL (from inception to January 2026), using Medical Subject Headings (MeSH) and free-text terms combining (A) HS, (B) palliative or supportive care, (C) quality of life or unmet needs, and (D) symptom-specific terms. The complete search strategy is presented in Table 2.

**Table 2:** Search strategy HRQoL, health-related quality of life; QoL, quality of life

Concept	Search Terms
Condition	"hidradenitis suppurativa" OR "acne inversa" OR "HS" OR "Verneuil disease"
Palliative/Supportive Care	"palliative care" OR "supportive care" OR "end-of-life" OR "symptom management" OR "comfort care" OR "hospice"
Quality of Life/Unmet Needs	"quality of life" OR "QoL" OR "HRQoL" OR "unmet needs" OR "patient-reported outcomes" OR "patient burden"
Symptom-Specific Terms	"pain" OR "fatigue" OR "depression" OR "anxiety" OR "sexual dysfunction" OR "wound" OR "drainage" OR "odour" OR "odor" OR "pruritus" OR "itch"
Databases Searched	MEDLINE (via PubMed), Web of Science, Cochrane CENTRAL
Date Range	Inception to January 2026

Study Selection, Extraction, and Quality Assessment

Two reviewers independently screened all titles/abstracts and full texts, with a third adjudicating disagreements. Data were extracted using a standardized, piloted form that collected the study design, population characteristics, outcome measures, scale properties, key quantitative findings (including measures of variance), unmet needs, and barriers to care. Quality was assessed using Joanna Briggs Institute (JBI) Prevalence Checklists (cross-sectional surveys), the Newcastle-Ottawa Scale (NOS) (observational/registry studies), and the Critical Appraisal Skills Programme (CASP) Quantitative Checklist (psychometric randomized controlled trial (RCT) analysis). A domain-based narrative synthesis framework was applied, with findings organized under pre-specified palliative care domains (physical symptoms, psychological burden, social and occupational impact, sexual health, HRQoL, and diagnostic delay/care access). Statistical heterogeneity in study design, population, and outcome measures precluded meta-analysis.

Results

Included Studies

**Figure 1:**
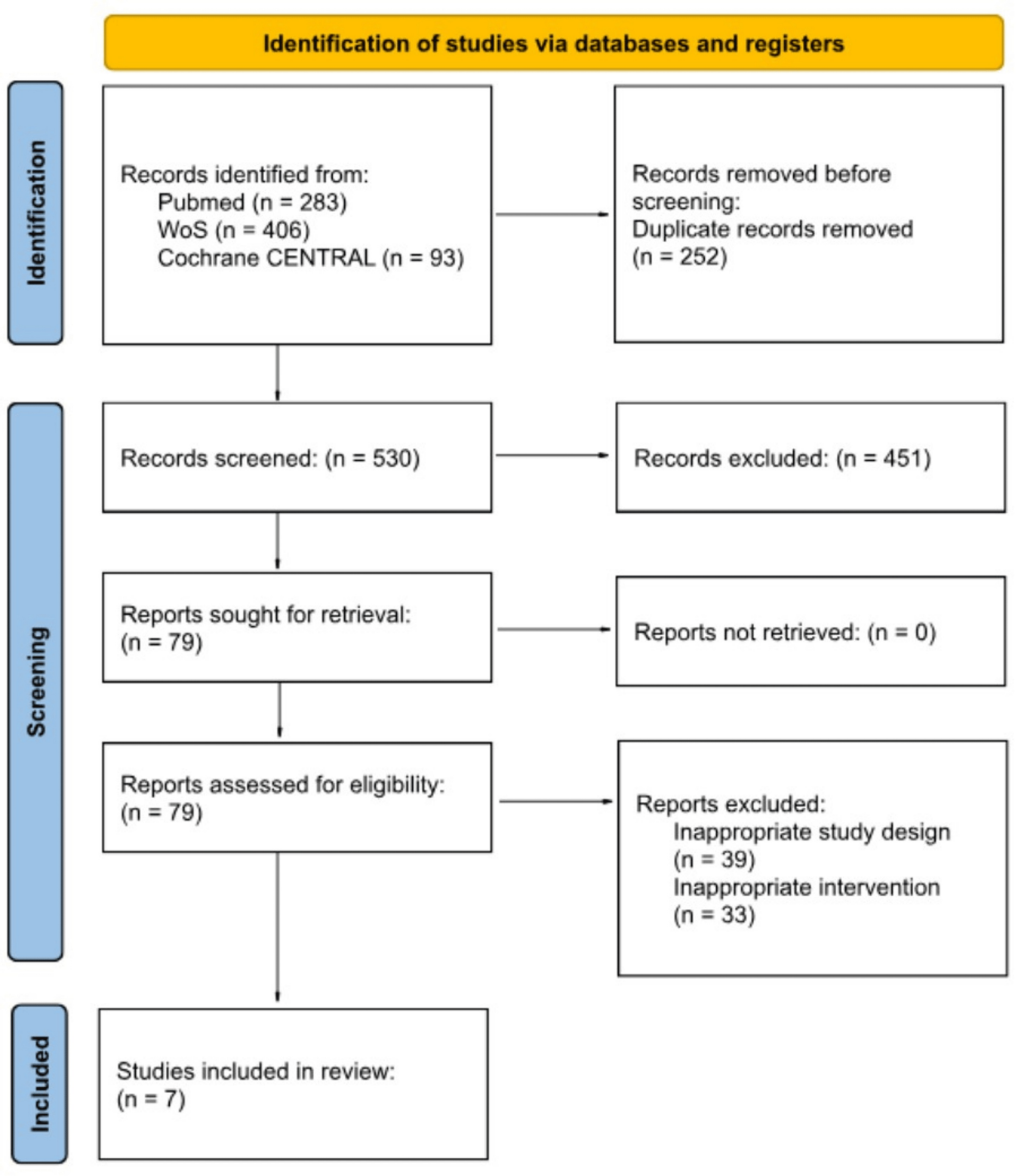
Flowchart of the reviewed studies according to PRISMA PRISMA, preferred reporting items for systematic reviews and meta-analyses

Seven studies published between 2020 and 2025 were included from the screened records (Figure 1): four cross-sectional surveys or retrospective analyses [11-14], one prospective observational registry [15], one single-center observational study [16], and one psychometric analysis of pooled phase III RCT data [17]. Studies spanned 27 countries; sample sizes ranged from 77 to 1,787. Participants were predominantly female (56.6-84.9%) with mean ages ranging from 33 to 39 years. Two of the included publications [11,14] drew on overlapping populations from the same Adelphi Real World Disease Specific Programme dataset (N = 1,787) but addressed non-overlapping research questions: Ingram et al. reported severity-stratified whole-cohort unmet needs [14], while Jaleel et al. reported racial/ethnic disparities in the patient journey [11]. To avoid double-counting, no quantitative estimate cited in this review is derived from both publications. Table 3 further demonstrates characteristics of the included studies.

**Table 3:** Characteristics of the included studies Jaleel et al. [11] and Ingram et al. [14] analyze overlapping populations from the same Adelphi Real World Disease Specific Programme dataset but report on non-overlapping research questions (racial/ethnic disparities and overall unmet clinical need, respectively); each data point cited in this review draws on one publication only. HS, hidradenitis suppurativa; DSP, disease-specific programme; HS-PGA, hidradenitis suppurativa physician global assessment; IHS4, International Hidradenitis Suppurativa Severity Score System; RCT, randomized-controlled trial

Study	Design/Period	Countries/Setting	N	Population & Disease Severity
Jaleel et al. (2024) [11]	Cross-sectional survey (Adelphi DSP™), Nov 2020-Apr 2021	France, Germany, Italy, Spain, UK, USA	N = 1,787 (312 dermatologists)	Mean age = 34.4 ± 12.2 yr; 57.6% female; 22.3% racial/ethnic minority. Hurley I = 54.7%; II 37.0%; III 8.3%. Mean flares = 1.9-2.2/yr
Garg et al. (2020) [12] Global VOICE	Prospective patient survey, Oct 2017-Jul 2018	14 countries (Europe 55%; N. America 38%); 27 HS referral centers	N = 1,299 complete cases	Mean age at diagnosis = 30.7 ± 10.9 yr; 84.9% female; 80.6% White. All Hurley stages. Mean diagnostic delay = 10.2 ± 8.9 yr; 14.5% work-disabled
Kimball et al. (2020) [15] UNITE Registry	Prospective observational registry (baseline analysis), Oct 2013-Dec 2015	12 countries; 73 sites (incl. Europe, N. America, Australia)	N = 529 adults + 65 adolescents	Adults: mean = 38.6 ± 12.6 yr; 68.6% female. Hurley I 5.3%; II 65.4%; III 29.3%. Only 4.5% complete disease control
Gergely et al. (2020) [13]	Cross-sectional, multicentre (psychometric validation), Sep 2017-Oct 2019	Hungary (3 academic centers)	N = 200 consecutive adults	Mean = 37.1 ± 12.4 yr; 61.5% male (atypical); 81.2% overweight/obese. HS-PGA moderate-very severe: 74.3%
Quinto et al. (2021) [16]	Cross-sectional, single center (sexual health), Jan 2018-Feb 2019	Italy; dedicated HS clinic	N = 77 adults	Mean = 33.4 ± 10.6 yr; 64.9% female. Hurley I 32.9%; II 59.2%; III 7.9%. IHS4 severe in 52.6%. Mean disease duration = 11.2 ± 8.2 yr
Ingram et al. (2022) [14]	Cross-sectional survey (Adelphi DSP™; same dataset as [11], different analysis), Nov 2020-Apr 2021	France, Germany, Italy, Spain, UK, USA	N = 1,787, Patient self-completion: 33.1%	Mean = 34.4 ± 12.2 yr; 57.6% female. Physician-judged: mild 66.0%; moderate 29.3%; severe 4.7%. >70% had moderate/severe disease at first diagnosis
Kirby et al. (2025) [17] BE HEARD I & II	Secondary psychometric analysis, pooled phase III RCT data (NCT04242446; NCT04242498)	Multinational: N. America = 38%; W. Europe = 29%; C./E. Europe = 26%; Asia/Australia = 7%	N = 1,010 (BE HEARD I: 502; II: 508)	Mean = 36.7 ± 12.2 yr; 56.6% female. All Hurley II (55.6%) + III (44.4%). Mean disease duration = 8.0 ± 7.8 yr

Physical Symptom Burden

Pain was the most consistently documented palliative need (Table 4). In the Global VOICE project (n = 1,299, 14 countries), 61.4% (n = 798) of patients rated HS-related pain as moderate or higher (numeric rating scale (NRS): ≥5/10) and 4.5% (n = 59) described the worst possible pain, with a mean NRS of 5.0 ± 2.8 [12]. People from racial and ethnic minority groups (PREG) reported significantly higher pain than White patients (3.3 ± 2.6 versus 2.5 ± 2.2; p = 0.011) [11]. In the UNITE registry (n = 529 adults; 73 sites; 12 countries), approximately 30% (n ≈ 156/521) of adults rated each Hidradenitis Suppurativa Symptom Assessment (HSSA) symptom item ≥7 (substantially burdensome), with tenderness (6.0 ± 3.4), redness (5.5 ± 3.3), and swollenness (5.3 ± 3.5) as the highest [15]. Despite active dermatologist management, pain or discomfort persisted in 49.5% (n = 885/1,787) of all patients in Ingram et al.'s real-world survey, rising to 81.2% (n = 69/85) in those with severe disease [14].

**Table 4:** Palliative and supportive care needs by domain: outcome measures and key findings PREG, people from racial/ethnic minority groups; NRS, Numeric Rating Scale; OR, odds ratio; HSSA, HS symptom assessment; HSIA, HS impact assessment; HiSQOL, HS quality-of-life questionnaire; DLQI, Dermatology Life Quality Index; EQ-5D-5L, EuroQol 5-Dimension 5-Level; GHQ-12, General Health Questionnaire; BMI, body mass index; HADS, Hospital Anxiety and Depression Scale; SDQ, Sexual Dysfunction Questionnaire; HS-PtGA, patient global assessment of HS; WPAI, work productivity and activity impairment; IHS4, International HS Severity Score System

Outcome Measure/Tool	Study	N	Key Finding
A. Physical Symptoms			
Pain (NRS 0-10)	[12]	1,299	9 in 10 reported HS-related pain; 61.4% NRS ≥5 (moderate–severe); mean = 5.0 ± 2.8. PREG worse than White patients (3.3 vs 2.5; p = 0.011) [11]
Physical symptom severity (HSSA, 0-10/item)	[15]	521	~30% rated each symptom ≥7 (substantially burdensome). Highest: tenderness 6.0 ± 3.4; redness 5.5 ± 3.3; pain 4.6 ± 3.4; drainage 3.7 ± 3.6
Drainage, odour, fatigue (patient self-report)	[12]	1,299	Drainage 71.8%; fatigue 61.0%; malodorous discharge 53.8% in prior week. Fatigue 30.6% in severe patients [14]
Persistent symptoms despite treatment (physician report)	[14]	1,787	Pain/discomfort 49.5%; inflammation 46.1%; drainage 26.5%; low mood 14.5%. Severe patients: pain 81.2%; malodorous drainage 60.0%
B. Psychological Burden			
Depression and anxiety (self-reported comorbidity)	[12]	1,299	Depression 35.8%; anxiety 36.2% (most frequent comorbidities). Suicidal ideation 7.9%; suicidal attempt 4.2%; substance use 3.6%
HADS anxiety/depression (≥11 = probable case)	[15]	508	Probable anxiety = 31.7%; probable depression: 19.3%. Mean HADS-Anxiety = 8.6 ± 4.5 (borderline range). Worsens with Hurley stage (depression: I 14.3% → III 25.0% probable)
Emotional burden vs. physical (Skindex-16 subscales)	[13]	200	Emotions subscale = 64.55 ± 29.28 significantly exceeded functioning (49.40) and symptoms (46.74). EQ-5D-5L anxiety/depression problems: 51%
Minor psychiatric disorder (GHQ-12 ≥4); high anxiety (HADS ≥11)	[16]	77	GHQ-12 caseness = 49.3%. HADS-Anxiety positive = 39.4%. Psychological factors are stronger predictors of sexual impairment than clinical severity
C. Social & Occupational Impact			
Work disability and employment (employment status; WPAI:SHP)	[12,15]	1,299/529	14.5% work-disabled [12]; only 60% adults employed [15]. Presenteeism: 63.6% with some impairment (mean 43.7 ± 26.7%). Overall work impairment 48.9 ± 29.5%; activity impairment 41.1 ± 33.2%
Daily-life impact (6 domains: appearance, mood, relationships, etc.)	[14]	557-578	Most impacted: personal appearance/self-confidence (15.1% greatly affected); mood (12.7%); close relationships (10.2%). Severe patients: 53.3% greatly affected for mood and appearance
Racial/ethnic disparities in social outcomes (WPAI:GH)	[11]	551	PREG: activity impairment 27.0 ± 25.2% vs. White 20.0 ± 20.6% (p = 0.008). PREG less likely employed (57.7% vs. 69.1%, p < 0.001). More desire for physician support (p = 0.006)
D. Sexual Health			
Sexual dysfunction (SDQ ≥45) and hindered sexuality	[16]	74-77	Sexual dysfunction: 60.8%. Hindered sexuality: 61.8%. Desire largely preserved (64.3% medium/high dyadic desire). Clinical severity NOT associated with sexual outcomes
Predictors of sexual impairment (multivariate logistic regression)	[16]	77	Sexual dysfunction: GHQ ≥4 (OR 7.04, p = 0.003); low BMI (OR 3.96, p = 0.033). Hindered sexuality: severe skin symptoms (OR 3.67, p = 0.031); anxiety (OR 4.40, p = 0.021)
Sexual intimacy impact (HSIA items, NRS 0-10)	[15]	427-458	Desire to have sex: mean = 6.2 ± 4.0 (highest HSIA burden item). >45% rated desire and ability to have sex ≥7 (substantially burdensome). Emotional > mobility impact (5.7 vs. 3.7)
E. Health-Related Quality of Life			
DLQI (0-30); HS worse than comparator skin conditions	[14,15]	505/555	UNITE: mean DLQI = 12.6 ± 8.0 (large effect) - exceeds psoriasis (9.8-11.8) and atopic dermatitis (8.8). Real-world: only 36.1% mild patients had DLQI 0-1 (no effect); 66.7% of severe had DLQI 11-30
EQ-5D-5L utility index (0-1); comparison with other conditions	[13]	198	Mean EQ-5D-5L index = 0.76 ± 0.21. HS showed greater pain/discomfort impairment than psoriasis and pemphigus vulgaris. 77% had pain or discomfort
HiSQOL (0-68)	[17]	997	Baseline mean = 25.2 ± 13.4. HiSQOL weakly correlated with IHS4 (r < 0.30)
F. Diagnostic Delay & Care Access			
Diagnostic delay (patient-reported timeline)	[11,12]	1,299/564	Mean delay 10.2 ± 8.9 yr (onset to diagnosis) [12]; 63.7% visited physician ≥5 times before diagnosis. Access rated difficult: 37.0%. ED visits ≥5 times: 18.3%. PREG delayed 2× longer [11]
Disease severity at diagnosis vs. at sampling	[14]	1,787	>70% presented with moderate/severe disease at first diagnosis. 92.9% of severe patients had scarring at sampling. 17.1% had unstable/deteriorating disease in prior 12 months
G. Treatment Satisfaction & Unmet Need			
Treatment dissatisfaction; disease control (HS-PtGA)	[12,15]	1,299/514	Dissatisfied with medical treatment: 45.9% [12]. Complete disease control (HS-PtGA = 0): only 4.5% adults [15]; 4.9% of severe patients in remission [14]. Low optimism for control within 3 months: 45.9%

Drainage was reported by 71.8% (n = 933/1,299), fatigue by 61.0% (n = 793/1,299), and malodorous discharge by 53.8% (n = 699/1,299) during the preceding week in Global VOICE [12]. No included study evaluated fatigue using a validated instrument, representing a measurement gap.

Psychological Burden

Psychiatric morbidity was near-universal (Table 4). Global VOICE reported depression in 35.8% (n = 465/1,299) and anxiety in 36.2% (n = 470/1,299) of participants, with suicidal ideation in 7.9% (n = 103/1,299) and suicidal attempt in 4.2% (n = 55/1,299) [12]. The UNITE registry, using the validated Hospital Anxiety and Depression Scale (HADS), found probable clinical anxiety (≥11) in 31.7% (n = 161/508) and probable clinical depression in 19.3% (n = 98/508) of adults; mean HADS-Anxiety was 8.6 ± 4.5 (borderline range), worsening with Hurley stage (probable depression: Stage I = 14.3% (n = 4/28) to Stage III = 25.0% (n = 37/148)) [15]. In Quinto et al. (n = 77), 49.3% (n = 37/75) met criteria for probable minor psychiatric disorder (General Health Questionnaire-12 (GHQ-12) ≥4) and 39.4% (n = 28/71) had clinically significant anxiety (HADS ≥11) [16].

Emotional burden regularly exceeded physical symptom burden across instruments. The Skindex-16 emotions subscale mean (64.55 ± 29.28) substantially exceeded both the functioning (49.40 ± 34.70) and symptoms (46.74 ± 29.36) subscales in the Hungarian multicentre validation study [13], and the Hidradenitis Suppurativa Impact Assessment (HSIA) emotional impact score (5.7 ± 3.4) exceeded the mobility impact score (3.7 ± 2.9) in the UNITE registry [15].

Social and Occupational Impact

Occupational disability was common across all studies (Table 4): 14.5% (n = 188/1,299) of Global VOICE participants were unable to work due to HS, and 9.6% (n = 125/1,299) were unemployed and seeking work, despite the majority being of working age [12]. In UNITE, only 60% (n = 317/529) of adults with HS were employed; 63.6% (n = 202/317) of those employed reported reduced on-the-job effectiveness (presenteeism; mean impairment 43.7 ± 26.7%), and mean overall activity impairment was 41.1 ± 33.2%, affecting 74.7% (n = 395/529) of adults to some degree [15]. Among all patients in the real-world survey, personal appearance and self-confidence were greatly affected in 15.1% (n = 87/577), mood in 12.7% (n = 73/576), and close personal relationships in 10.2% (n = 59/578); in those with severe disease, more than half were greatly affected across both mood and personal appearance [14]. Social withdrawal was rated as very much or extremely impacting life by 48.6% (n = 631/1,299) of Global VOICE respondents, and embarrassment about their condition was very much or extremely bothersome to 37.3% (n = 485/1,299) [12]. PREG faced compounded social and occupational disadvantage: lower employment rates (57.7% (n = 214/371) versus 69.1% (n = 890/1,288); p < 0.001), greater activity impairment (27.0 versus 20.0%; p = 0.008), and a significantly greater reported desire for physician support with disease management (p = 0.006) [11].

Sexual Health

Sexual health was among the most severely impacted and most underaddressed palliative care domains (Table 4). Using the validated Sexual Dysfunction Questionnaire, 60.8% (n = 45/74) of HS patients demonstrated clinical sexual dysfunction, and 61.8% (n = 47/76) reported hindered sexuality; medium or high sexual desire was retained by the majority (dyadic: 64.3%; solitary: 64.7%), consistent with HS disrupting function through pain, lesion location, and embarrassment rather than through loss of libido [16].

Multivariate regression identified psychological aspects, not clinical severity, as the primary drivers of sexual impairment. Probable minor psychiatric disorder (GHQ-12 ≥4) increased odds of sexual dysfunction (odds ratio (OR): 7.04; 95% confidence interval (CI): 1.93-25.7; p = 0.003) and low dyadic desire (OR: 5.02; 95% CI: 1.50-16.8; p = 0.009); clinically significant anxiety predicted hindered sexuality (OR: 4.40; 95% CI: 1.25-15.5; p = 0.021). Hurley stage, IHS4, and Sartorius score were not associated with any sexual outcome [16]. Corroborating this, sexual intimacy proved the highest-burden HSIA item in UNITE (desire to have sex: 6.2 ± 4.0/10; >45% rated ≥7) and among the most severely impacted HiSQOL domains in Global VOICE [12,15].

Health-Related Quality of Life

HRQoL was profoundly impaired across all studies, consistently exceeding burden in comparator dermatological conditions (Table 4). In UNITE, the mean Dermatology Life Quality Index (DLQI) was 12.6 ± 8.0 [15], and only 36.1% (n = 137/379) of mild patients had a DLQI of 0-1 (no impact on quality of life) [14]. The EuroQol 5-Dimension 5-Level (EQ-5D-5L) demonstrated greater pain/discomfort impairment in HS than in psoriasis or pemphigus vulgaris, with a mean utility index of 0.76 ± 0.21 [13].

The Hidradenitis Suppurativa Quality of Life (HiSQoL) questionnaire was validated using blinded pooled data from 1,010 patients enrolled in two international phase III RCTs (BE HEARD I and II) [17]. The Hidradenitis Suppurativa Core Outcomes Set International Collaboration (HiSTORIC) has endorsed HiSQOL as the recommended HRQoL measure for routine HS clinical practice [18]. The weak correlation between HiSQOL scores and the physician-rated IHS4 at baseline (r < 0.30) confirmed that patient-reported quality of life and the clinician-assessed disease severity measure represent connected yet distinct constructs [17].

Diagnostic Delay, Treatment Satisfaction, and Unmet Need

Mean diagnostic delay from symptom onset ranged from 7.2 to 10.2 years across included studies; 63.7% (n = 827/1,299) of Global VOICE participants had visited a physician five or more times before receiving a correct diagnosis, 37.0% (n = 481/1,299) rated dermatology access as difficult or very difficult, and 18.3% (n = 238/1,299) had attended the emergency department (ED) five or more times for HS symptoms [12]. In the Ingram et al. real-world survey, over 70% (n = 1,315/1,787) of patients presented with moderate or severe disease at the time of their first formal HS diagnosis, and 92.9% (n = 79/85) of those with severe disease had acquired irreversible scarring by the time of sampling [14]. The consequences for palliative care are direct: the longer a correct diagnosis is delayed, the greater the accumulation of irreversible disease burden, scarring, and associated psychological distress. PREG experienced significantly longer delays both from symptom onset to first consultation and from consultation to correct diagnosis, alongside disproportionately higher rates of misdiagnosis with boils rather than HS [11].

Treatment dissatisfaction remained widespread: 45.9% (n = 596/1,299) of Global VOICE respondents were dissatisfied with medical treatment, 34.6% (n = 449/1,299) with procedural treatments, and 45.9% (n = 596/1,299) expressed low optimism for symptom control within three months, with perceived poor efficacy (42.1%) as the predominant reason [12]. Only 4.5% (n = 24/529) of adults in UNITE achieved complete disease control (Patient Global Assessment of HS (HS-PtGA) = 0), with 67.9% (n = 359/529) reporting limited or uncontrolled disease [15]. In the real-world survey, 4.9% (n = 4/82) of patients with severe disease were in remission at the time of sampling [14]. The persistence of significant symptoms despite active dermatologist management in the majority of patients constitutes a palliative care imperative: when disease-modifying treatment is insufficient, symptom management, psychological support, and quality-of-life optimization become primary therapeutic goals rather than secondary considerations. Unmet needs, barriers, and evidence gaps across all domains are synthesized in Table 5; of particular note, caregiver burden, spiritual and existential needs, and advance care planning were entirely absent from the evidence base.

**Table 5:** Unmet palliative and supportive care needs, barriers, and research priorities Unmet needs were synthesized from all seven included studies. Research gaps identified by the review team. CBT, cognitive behavioral therapy; PREG, people from racial/ethnic minority groups; RCT, randomized controlled trial

Palliative Care Domain	Unmet Needs (key evidence)	Barriers to Supportive Care	Evidence Gaps/Research Priorities
Physical Symptom Management (pain, drainage, odour, fatigue)	No HS-specific pain management protocol. Substance use disorder ~4% (pain-coping signal). Drainage, odor, and fatigue prevalent but without palliative protocols. Symptoms persist in ~50% of patients despite active treatment [14].	Absence of validated HS analgesic pathways. Limited wound care nursing protocols. Physician-rated severity underestimates patient burden (r < 0.30 with HRQoL) [13,17]. No fatigue-specific screening in HS.	No RCTs of pain management strategies in HS. No evaluation of wound care or odor management protocols. Fatigue as a standalone palliative need unstudied.
Psychological & Psychiatric Support (depression, anxiety, suicidality)	Depression 30-36%, anxiety 36-58%; suicidal ideation 7.9%; suicidal attempt 4.2% - largely unscreened in dermatology [12,15]. Emotional burden exceeds physical symptom burden [13]. Psychological distress not predicted by Hurley stage.	No integrated mental health pathways in HS dermatology settings. Stigma prevents disclosure. Clinical severity scores miss psychological need. Only 6% of registry patients had physician-documented psychiatric comorbidity management [15].	No RCTs of CBT, mindfulness, or acceptance therapy in HS. No longitudinal suicidality data. No evaluation of distress screening tools in HS outpatient settings.
Social & Occupational Support (employment, relationships, stigma)	14.5% work-disabled; only 60% employed; 74.7% report activity impairment [12,15]. Relationships, appearance, and self-confidence most impacted daily-life domains [14]. Racial/ethnic minorities face compounded social disadvantage [11].	Disability/occupational rehabilitation rarely addressed in dermatology. Stigma around malodorous discharge hinders social participation. 37% rate dermatology access as difficult [12]. Cultural/language barriers delay care-seeking in PREG [11].	No occupational rehabilitation studies in HS. Social support needs of caregivers and family members not studied. No evaluation of equity-focused care models.
Sexual Health (functioning, desire, intimacy)	Sexual dysfunction 60.8%; hindered sexuality 61.8% [16] - rarely assessed in consultations. Psychological factors (not severity) drive impairment; targeted intervention likely effective. Lesion location and pain impair function despite preserved desire.	Sexual history rarely taken in dermatology. Shame and partner disclosure anxiety prevent reporting. No HS sexual health guidelines. SDQ/SDI-2 not yet in routine use.	No RCTs of sex therapy or sexual health counselling in HS. Partner perspectives unstudied. No longitudinal sexual health trajectory data.
Diagnostic Equity & Access	Mean delay 10.2 ± 8.9 yr [12]; >70% with moderate/severe disease at first diagnosis [14]. PREG experience 2× longer diagnostic delay and disproportionate misdiagnosis [11]. ED overuse (18.3% with ≥5 visits) signals care access failure.	Under-recognition in skin of colour. Medical mistrust in PREG. Inadequate primary-care HS education. No point-of-care diagnostic aids. Limited dermatology capacity.	No interventions specifically targeting diagnostic delay in HS. Impact of delay on palliative burden unstudied. Healthcare equity interventions for PREG absent from literature.
Caregiver, Spiritual & Existential Needs; Advance Care Planning	Caregiver burden, advance care planning, and spiritual/existential needs are entirely absent from the HS evidence base. None of the seven included studies addressed these domains despite their centrality to palliative care frameworks.	No validated caregiver burden instruments applied in HS. Advance care planning not considered relevant by treating dermatologists. Spiritual care not integrated into any HS care model.	Primary qualitative and quantitative research needed on caregiver burden, spiritual distress, existential suffering, and advance care planning in HS. Service models for non-malignant palliative care need to be adapted.

Validated Outcome Measures

In addition to the domain-specific findings above, a cross-cutting analysis of the measurement instruments used across the included studies was undertaken to identify validated tools applicable to palliative care assessment in HS. Twelve such measures were identified. These encompass HS-specific HRQoL (HiSQOL [17,19], HSSA, HSIA [15]), dermatology-specific HRQoL (DLQI, DLQI-R, Skindex-16 [13]), generic HRQoL and utility measurement (EQ-5D-5L [13]), psychological burden (HADS [15,16], GHQ-12 [16]), functional and occupational impairment (Work Productivity and Activity Impairment: General Health (WPAI: GH), Work Productivity and Activity Impairment: Specific Health Problem (WPAI: SHP) [11,15]), and sexual health (Sexual Dysfunction Questionnaire (SDQ), Sexual Desire Inventory-2 (SDI-2) [16]). The DLQI-Relevant (DLQI-R) (a modified scoring of the DLQI for patients who mark items as ‘not relevant’) demonstrated marginally better known-groups validity than the standard DLQI in HS, with a relative efficiency of 1.076 [13]. The EQ-5D-5L enables computation of quality-adjusted life years (QALYs), making it particularly valuable for health economic evaluation of potential palliative care interventions. A consistent finding was the weak-to-moderate correlation between clinician-rated severity scores and all patient-reported measures [13], highlighting that palliative care needs assessment is better operated independently of, and not derived from, clinical staging.

Quality Assessment

Quality appraisal results are presented in Table 6. Two studies were rated low risk of bias [13,17], one low-to-moderate risk [15], and four moderate risk [11,12,14,16]. Recurring limitations across survey studies included cross-sectional design, selection bias in registry enrolment, and lack of multivariate adjustment for confounding. Commercial or pharmaceutical funding was present in five of seven studies.

**Table 6:** Quality appraisal and risk of bias Assessed using the Joanna Briggs Institute (JBI) Prevalence Checklist (cross-sectional studies), Newcastle-Ottawa Scale (observational/registry), and CASP Quantitative Checklist (psychometric RCT analysis). -, not applicable or not reported; N/A, not applicable by design

Study (Appraisal Tool)	Sample Representativeness	Outcome Measurement	Confounding Adjustment	Data Completeness	Overall Risk of Bias
Jaleel et al. [11] (JBI Prevalence)	Low	Low	Moderate	Low	Moderate
Garg et al. [12] (JBI Prevalence)	Moderate	Low	-	Low	Moderate
Kimball et al. [15] (NOS)	Low	Low	-	Low	Low-Moderate
Gergely et al. [13] (NOS)	Low	Low	Low	Low	Low
Quinto et al. [16] (NOS)	Moderate	Low	Low	Low	Moderate
Ingram et al. [14] (JBI Prevalence)	Low	Low	-	Moderate	Moderate
Kirby et al. [17] (CASP)	Low	Low	N/A	Low	Low

Discussion

Principal Findings

This is, to our knowledge, the first systematic review of palliative and supportive care needs in HS. Synthesizing data from seven studies across 27 countries and more than 5,000 patient encounters, we demonstrate that the palliative burden of advanced HS is extraordinary, multidimensional, and substantially unmet. In each of the domains examined, physical symptoms, psychological morbidity, occupational disability, sexual health, HRQoL, and care access, the evidence documents impairment that meets or exceeds that of conditions for which integrated supportive care is already a standard of care expectation. The convergence of findings from registry data, multinational patient surveys, and psychometric analyses across several settings strengthens confidence in these conclusions despite the cross-sectional nature of the included studies. Caregiver burden, spiritual needs, and advanced care planning remain understudied, representing significant evidence gaps.

The Case for Palliative Care Integration

As detailed in the Results, complete disease control remains the exception rather than the rule, and psychiatric morbidity is near-universal. The consistently weak correlation between clinician-rated disease severity and patient-reported outcomes [13,17] illustrates a fundamental issue: standard dermatological assessment captures inflammatory lesion burden, not the full spectrum of patients' suffering. Palliative care assessment using validated, patient-focused instruments must therefore operate alongside, not be derived from, clinical severity scoring.

Pain Management and Wound Care Gaps

Pain is one of the top patient-identified priorities in the international HiSTORIC Delphi consensus [20], yet no validated HS-specific analgesic protocol exists [21,22], and approximately 4% (n = 47/1,299) of patients develop substance use disorder, which might suggest inadequate pain management [12,23]. Wound-related symptoms, drainage, malodorous discharge, and associated wound burden constituted a separate and clinically significant palliative domain (Table 4). These symptoms are dignity-threatening, can preclude social participation and intimate relationships, and are rarely addressed by HS-specific clinical guidelines despite having established management options from palliative wound care practice. Fatigue has likewise not been measured using a validated instrument in any included study, representing both a measurement gap and an understudied therapeutic target.

Psychological and Sexual Health

Rates of depression, anxiety, and suicidal ideation in HS are severe enough to mandate formal integration of psychological care into HS management. Qualitative evidence identifies the causes driving distress in HS - shame and stigmatization arising from malodorous discharge and visible lesions, progressive loss of self-efficacy, and the impact of an unpredictable disease course on future planning [24,25] - and provides targets for cognitive behavioral and acceptance-based interventions, neither of which has been evaluated in an HS RCT. The HADS provides a validated and efficient first-line screening instrument for clinical use, with established cutoffs for borderline and probable clinical anxiety and depression [15].

For sexual health, the finding that psychological factors, not clinical disease severity, are the primary predictors of dysfunction [16] carries important clinical implications: it indicates that dermatological treatment intensification alone is insufficient to restore sexual well-being and that targeted psychological and sexual health counseling is likely to yield meaningful benefit. The SDQ and SDI-2 provide validated instruments for sexual health screening, and the pattern of preserved desire with impaired functioning suggests that patients are motivated to engage with therapeutic support if it is offered.

Diagnostic Delay

The diagnostic delay is one of the most consequential findings in this review [11,12]. Prolonged undiagnosed disease allows progression through Hurley stages, accumulation of irreversible scarring, and compounding of psychological and social distress that might have been partially mitigated by earlier diagnosis and intervention [26,27]. PREG face approximately twice the diagnostic delay compared to White patients [11], consistent with broader evidence of systemic racial disparities in dermatological recognition, access, and management [28].

Limitations

The included studies vary in severity composition: five enrol predominantly advanced-disease cohorts (≥67% Hurley Stages II-III or equivalent), while two qualify based on a substantial advanced-disease subgroup within a mixed-severity cohort. Although severity-stratified estimates were extracted preferentially, residual heterogeneity in baseline severity should be borne in mind when interpreting cross-study comparisons. The evidence base comprises entirely cross-sectional or retrospective studies and one psychometric analysis; no prospective longitudinal data or palliative care intervention studies in HS were identified. Studies are geographically concentrated in Europe and North America, limiting generalizability. Caregiver burden, spiritual and existential needs, and advanced care planning remain unstudied.

## Conclusions

Adults with advanced HS carry a palliative and supportive care burden that is multidimensional, substantial, and poorly met by current care frameworks. Intractable pain, severe psychiatric morbidity, sexual dysfunction, and occupational disability collectively constitute profound palliative care needs. A long diagnostic delay serves as a structural failure that prolongs this unmet symptom burden and compounds psychosocial distress over time. Racial and ethnic minorities face additional difficulties.

Priorities for clinical practice arising from this review include the adoption of validated palliative care assessment tools in specialist HS consultations; formal psychological screening; sexual health history-taking in the specialist review; and multidisciplinary team input encompassing pain management, psychological, occupational, and sexual health expertise. Priorities for research include palliative care intervention trials in HS; longitudinal studies tracking palliative burden over time; investigation of caregiver needs and family impact; and qualitative and quantitative research on spiritual and existential dimensions of suffering in advanced HS.
